# Kernel learning for robust dynamic mode decomposition: linear and nonlinear disambiguation optimization

**DOI:** 10.1098/rspa.2021.0830

**Published:** 2022-04

**Authors:** Peter J. Baddoo, Benjamin Herrmann, Beverley J. McKeon, Steven L. Brunton

**Affiliations:** ^1^ Department of Mathematics, Massachusetts Institute of Technology, Cambridge, MA 02139, USA; ^2^ Department of Mechanical Engineering, University of Chile, Beauchef 851, Santiago, Chile; ^3^ Graduate Aerospace Laboratories, California Institute of Technology, Pasadena, CA 91125, USA; ^4^ Department of Mechanical Engineering, University of Washington, Seattle, WA 98195, USA

**Keywords:** machine learning, kernel methods, system identification, modal decomposition

## Abstract

Research in modern data-driven dynamical systems is typically focused on the three key challenges of high dimensionality, unknown dynamics and nonlinearity. The dynamic mode decomposition (DMD) has emerged as a cornerstone for modelling high-dimensional systems from data. However, the quality of the linear DMD model is known to be fragile with respect to strong nonlinearity, which contaminates the model estimate. By contrast, sparse identification of nonlinear dynamics learns fully nonlinear models, disambiguating the linear and nonlinear effects, but is restricted to low-dimensional systems. In this work, we present a kernel method that learns interpretable data-driven models for high-dimensional, nonlinear systems. Our method performs kernel regression on a sparse dictionary of samples that appreciably contribute to the dynamics. We show that this kernel method efficiently handles high-dimensional data and is flexible enough to incorporate partial knowledge of system physics. It is possible to recover the linear model contribution with this approach, thus separating the effects of the implicitly defined nonlinear terms. We demonstrate our approach on data from a range of nonlinear ordinary and partial differential equations. This framework can be used for many practical engineering tasks such as model order reduction, diagnostics, prediction, control and discovery of governing laws.

## 1. Introduction

Discovering interpretable patterns and models from high-dimensional data is one of the principal challenges of scientific machine learning, with the potential to transform our ability to predict and control complex physical systems [1]. The current surge in the quality and quantity of data, along with rapidly improving computational hardware, has motivated a wealth of machine learning techniques that uncover such patterns for dynamical systems. Successful recent methods include the dynamic mode decomposition (DMD) [2–5] and extended DMD (eDMD) [6,7], sparse identification of nonlinear dynamics (SINDy) for ordinary and partial differential equations [8,9], genetic programming for model discovery [10], physics-informed neural networks (PINNs) [11], Lagrangian neural networks [12], time-lagged autoencoders [13], operator theoretic methods [14] and operator inference [15]. Techniques based on generalized linear regression, such as DMD and SINDy, are widely used because they are computationally efficient, require less data than neural networks, are highly extensible and provide interpretable models. However, these approaches are either challenged by nonlinearity (e.g. DMD) or do not scale to high-dimensional systems (e.g. SINDy). In this work, we present a machine learning algorithm that leverages sparse kernel regression to address both challenges, efficiently learning high-dimensional nonlinear models that admit interpretable spatio-temporal coherent structures and robust locally linear models.

A central goal of modern data-driven dynamical systems is to identify a model

1.1 $$\frac{d}{dt}x=F(x)=Lx+N(x),$$

that describes the evolution of the state of the system, x. Here, we explicitly indicate that the dynamics F have a linear L and nonlinear N contribution, although many techniques do not model these separately or explicitly. However, several approaches obtain interpretable and explicit models of this form. For example, DMD seeks a best-fit linear model of the dynamics, while SINDy directly identifies sparse nonlinear models of the form in (1.1).

Our approach synthesizes favourable aspects of several approaches mentioned above; however, it most directly complements and addresses the challenges of DMD for strongly nonlinear systems. The DMD was originally introduced by Schmid [2] in the fluid dynamics community as a method for extracting spatio-temporal coherent structures from high-dimensional data, resulting in a low-rank representation of the best-fit linear operator that maps the data forward in time [4,5]. The resulting linear DMD models have been used to characterize many systems in fluid mechanics, where complex flows admit dominant modal decompositions [16]. DMD has also been adopted in a wide range of fields beyond fluid mechanics, and much of its success stems from the formulation of DMD as a linear regression problem [4], based entirely on measurement data, resulting in several powerful extensions [5]. However, because DMD uses least-squares regression to find a best-fit linear model dx/dt≈Ax to the data, the presence of measurement noise [17], control inputs [18] and nonlinearity bias the regression. Mathematically, the noise, control inputs and nonlinearity may all be lumped into a forcing b

1.2 $$\frac{d}{dt}x=Lx+b\approx Ax.$$

**Figure 1.**
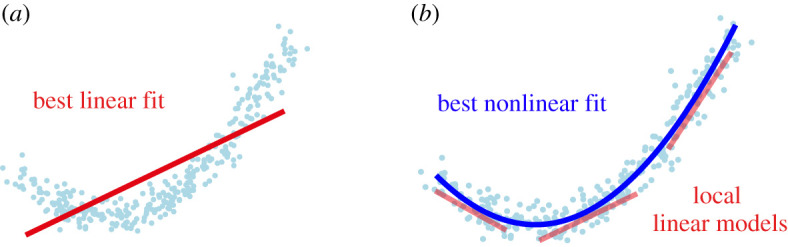
Learning regression models in linear (*a*) and nonlinear (*b*) feature spaces. Our approach disambiguates linear and nonlinear model contributions to accurately extract local linear models. (Online version in colour.)

The forcing b contaminates the linear model estimate, so A from DMD does not approximate the true linear contribution from L. It was recognized early on that the DMD algorithm was highly sensitive to noise [17], resulting in noise-robust variants, including forward backward and total least-squares DMD [17], optimized DMD [19] and DMD based on robust principal component analysis (PCA) [20]. Similarly, DMD with control [18] was introduced to disambiguate the effect of the linear dynamics from actuation. For statistically stationary systems with stochastic inputs, the spectral proper orthogonal decomposition [21] produces an optimal basis of modes to describe the variability in an ensemble of DMD modes [22]. The bias due to nonlinearity, shown in figure 1*a*, has been less thoroughly explored and is the topic of the present work.

Despite these challenges, DMD is frequently applied to strongly nonlinear systems, with theoretical motivation from Koopman operator theory [3,5,14,23]. Williams *et al.* [6] developed the eDMD, which augments the original state with nonlinear functions of the state to better approximate the nonlinear eigenfunctions of the Koopman operator for nonlinear systems. However, because this approach still fundamentally results in a linear model (in the augmented state), it also suffers from the same issues of not being able to handle multiple fixed points or attracting structures, and it also typically suffers from closure issues related to the irrepresentability of Koopman eigenfunctions. Delay embedding methods, such as Hankel DMD [24] and higher-order DMD [25], are effective for computing Koopman eigenfunctions but do not separate the linear and nonlinear mechanisms of the system. The SINDy [8] algorithm is a related regression approach to model discovery, which identifies a fully nonlinear model as a sparse linear combination of candidate terms in a library. While SINDy is able to effectively disambiguate the linear and nonlinear dynamics in (1.1), resulting in the ability to obtain de-biased locally linear models as in figure 1*b*, it only applies to relatively low-dimensional systems because of poor scaling of the library with state dimension.

### (a) Contributions of this work

**Figure 2.**
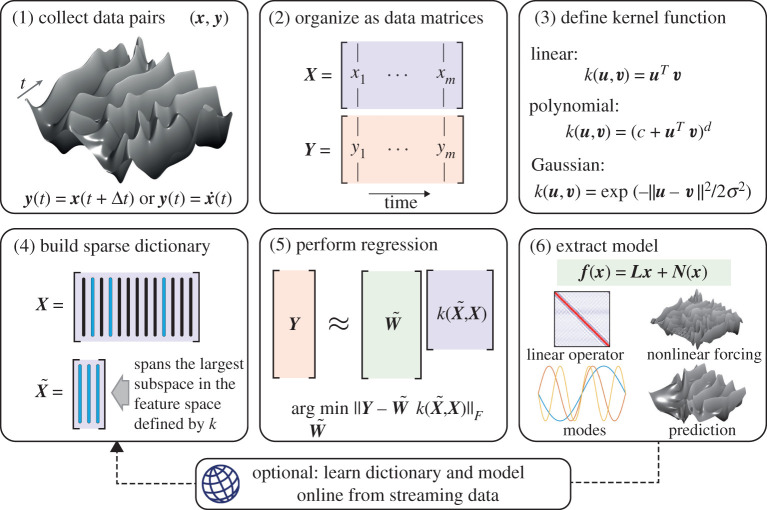
The linear and nonlinear disambiguation optimization (LANDO) framework. Training data in the form of snapshot pairs are collected from either simulation or experiment in (1). The data are organized into matrices in (2). In (3), an appropriate kernel is defined, which can be informed by expert knowledge of the underlying physics of the system or through cross-validation. In (4), a sparse dictionary of basis elements is constructed from the training samples, and in (5), the regression problem is solved. Finally, in (6), an interpretable model is extracted. (Online version in colour.)

In this work, we develop a custom kernel regression algorithm to learn accurate, efficient and interpretable data-driven models for strongly nonlinear, high-dimensional dynamical systems. This approach scales to very high dimensions, unlike SINDy, yet still accurately disambiguates the linear part of the model from the implicitly defined nonlinear dynamics. Thus, it is possible to obtain linear DMD models, local to a given base state, that are robust to strongly nonlinear dynamics. Our approach, referred to as the linear and nonlinear disambiguation optimization (LANDO) algorithm, may be viewed as a generalization of DMD that enables a robust disambiguation of the underlying linear operator from nonlinear forcings. The learning framework is illustrated in figure 2, and open-source code is available at www.github.com/baddoo/LANDO.

To achieve this robust learning, we improve upon several leading kernel and system identification algorithms. Recent works have successfully applied kernel methods [26,27] to study data-driven dynamical systems [7,28–31]. A key inspiration for the present work is kernel DMD (kDMD, [7]), which seeks to approximate the infinite-dimensional Koopman operator as a large square matrix evolving nonlinear functions of the original state. An essential difference between kDMD and the present work is that our goal is to implicitly model the (non-square) nonlinear dynamics in (1.1) in terms of the original state x, enabling the robust extraction of the linear component L, as opposed to analysing the Koopman operator over measurement functions. Further differences between LANDO and other DMD methods are elucidated in the electronic supplementary material, SI §C. In our work, we present a modified kernel recursive least-squares (KRLS) algorithm [32] to learn a nonlinear model that best characterizes the observed data. To reduce the training cost, which typically scales with the cube of the number of training samples for kernel methods, we use dictionary learning to iteratively identify samples that appreciably contribute to the dynamics. This dictionary learning approach may be seen as a sparsity promoting regularizer, significantly reducing the high condition number that is common with kernel methods, thus improving robustness to noise. We introduce an iterative Cholesky update to construct the dictionary in a numerically stable manner, significantly reducing the training cost while also mitigating overfitting. Similarly to KRLS, our model has the option of operating online by parsing data in a streaming fashion and exploiting rank-one matrix updates to revise the model. Therefore, our approach is also suitable for model order reduction in practical applications where data become available ‘on-the-fly’. Furthermore, we show how to incorporate partially known physics, or uncover unknown physics, by designing or testing tailored kernels, much as with the SINDy framework [8,33].

We demonstrate our proposed kernel learning approach on a range of complex dynamical systems that arise in the physical sciences. As an illustrative example, we first explore the chaotic Lorenz system. We also consider partial differential equations, using the LANDO framework to uncover the linear and nonlinear components of the nonlinear Burgers’, and Kuramoto–Sivashinsky (KS) equations using only nonlinear measurement data. The algorithm accurately recovers the spectrum of the linear operator for these systems, enabling linearized analyses, such as linear stability, transient growth and resolvent analyses [34,35], in a purely data-driven manner [36], even for strongly nonlinear systems. We also demonstrate the approach on a high-dimensional system of coupled nonlinear Kuramoto oscillators.

Whether the linear and nonlinear dynamics can be separated depends on both the underlying system and the available data. Our results indicate that the effectiveness of LANDO depends on the sampling rate, whether the data is near an attractor, and the amplitude of measurement noise. These issues are discussed throughout, but resolving them fully is the subject of ongoing work.

The remainder of the work is organized as follows. Section 2 provides a mathematical background overview of the DMD and kernel methods. Section 3 introduces our kernel learning procedure for dynamical systems, including the sparse dictionary learning with Cholesky updates. We demonstrate how to extract interpretable structures from these kernel models, such as robust linear DMD models, in §4. Results on a variety of nonlinear dynamical systems are presented in §5. Finally, §6 concludes with a discussion of limitations and suggested extensions of the method. The appendices (in the electronic supplementary material) explicate the connection between LANDO and DMD, demonstrate how to incorporate the effects of control, present the equations for online updating and investigate the noise sensitivity of the algorithm.

## 2. Problem statement and mathematical background

In this section, we will define our machine learning problem and review some relevant mathematical ideas related to DMD (§2a) and kernel methods (§2b).

We consider dynamical systems describing the evolution of an n-dimensional vector $x\in R^{n}$ that characterizes the state of the system. We will consider both continuous-time and discrete-time dynamics in this work. The dynamics may be expressed either in continuous time as

$$\frac{d}{dt}x(t)=F(x(t))$$

or in discrete time as

$$x_{j+1}=F(x_{j}).$$

For a given physical system, the continuous-time and discrete-time representations of the dynamics will correspond to different functions F, although we use the same function above for notational simplicity. In general, the dynamics may also vary in time and depend on control inputs u and parameters β; however, for simplicity, we begin with the autonomous dynamics above.

Our goal is to learn a tractable representation of the dynamical system $F:R^{n}\to R^{n}$ that is both accurate and interpretable, informing tasks such as physical understanding, diagnostics, prediction and control. We suppose that we have access to a training set of data pairs $\left\{(x_{j},y_{j})\in R^{n}\times R^{n}|j=1,\ldots ,m\right\}$, which are connected through the dynamics by

2.1 $$y_{j}=F(x_{j}).$$

If the dynamics are expressed in continuous time then $y_{j}={\dot{x}}_{j}$ where the dot denotes differentiation in time, and if the dynamics are expressed in discrete time then $y_{j}=x_{j+1}$. The discrete-time formulation is more common, as data from simulations and experiments are often sampled or generated at a fixed sampling interval Δt, so $x_{j}=x(j\Delta t)$. However, this work applies to both discrete and continuous systems, and the only practical difference arises in the eigenvalues of linearized models.

The training data correspond to m snapshots in time of a simulation or experiment. For ease of notation, it is typical to arrange the samples into snapshot data matrices of the form

2.2 $$X=\left[\begin{array}{cccc} | & | & & |\\ x_{1} & x_{2} & \cdots & x_{m}\\ | & | & & | \end{array}\right]\;\mathrm{and}\;Y=\left[\begin{array}{cccc} | & | & & |\\ y_{1} & y_{2} & \cdots & y_{m}\\ | & | & & | \end{array}\right].$$

In many applications, the state dimension is much larger than the number of snapshots, so m≪n. For example, the state may correspond to a fluid velocity field sampled on a discretized grid.

Our machine learning problem consists of finding a function f that suitably maps the training data given certain generalizability, interpretability and regularity qualifications. In our notation, F is the true function that generated the data whereas f is our model for F; it is hoped that F and f share some meaningful properties. The function f is typically restricted to a given class of models (e.g. linear, polynomial, etc.), so that it may be written as the expansion

2.3 $$f(x)=\sum_{j=1}^{N}\xi_{j}\phi_{j}(x)⟹f(x)=\Xi \phi (x).$$

Here, ϕ describes the feature library of N candidate terms that may describe the dynamics, and Ξ contains the coefficients that determine which model terms are active and in what proportions.

Mathematically, the optimization problem to be solved is

2.4 $$\underset{\Xi }{\mathrm{argmin}}||Y-\Xi \phi (X)|{|}_{F}+\lambda R(\Xi ),$$

where $||\cdot |{|}_{F}$ is the Frobenius norm. The first term in (2.4) corresponds to the error between training samples and our model prediction, whereas the second term λR(Ξ) is a regularizer. For example, in SINDy, the feature library ϕ will typically include linear and nonlinear terms, and the regularizer will involve the number of non-zero elements $||\Xi |{|}_{0}$, which may be relaxed to the 1-norm $||\Xi |{|}_{1}$. In DMD, the features ϕ will simply contain the state x, Ξ will be the DMD matrix A, and instead of a regularizer R(Ξ), the minimization is constrained so that the rank of A=Ξ is less than or equal to r. Similarly, in eDMD, the feature ϕ will include nonlinear functions of the state, and the minimization is modified to ${\mathrm{argmin}}_{\Xi }||\phi (Y)-\Xi \phi (X)|{|}_{F}+\lambda R(\Xi )$, resulting in a Ξ that is a large square matrix evolving the nonlinear feature space forward in time.

For even moderate state dimensions n and feature complexity, such as the monomials of order d, the feature library of ϕ becomes prohibitively large and the optimization in (2.4) is intractable. This scaling issue is the primary challenge in applying SINDy to high-dimensional systems. Instead, it is possible to rewrite the expansion (2.3) in terms of an appropriate kernel function k as

2.5 $$f(x)=\sum_{j=1}^{m}w_{j}k(x_{j},x)⟹f(x)=Wk(X,x).$$

In this case, the sum is over the number of snapshots m instead of the number of library elements N, dramatically improving the scaling. The optimization in (2.4) now becomes

2.6 $$\underset{W}{\mathrm{argmin}}||Y-Wk(X,X)|{|}_{F}+\lambda R(W).$$

**Figure 3.**
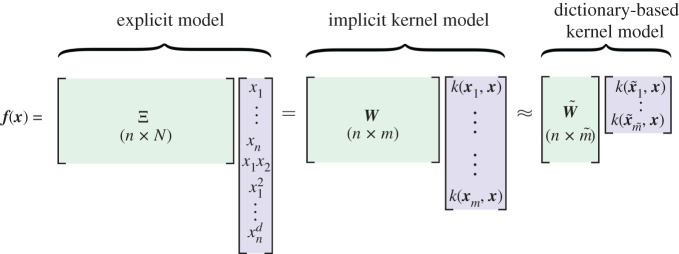
Schematic relationships between different models for $N\gg n,m\gg \tilde{m}$. An explicit model (e.g. SINDy) produces explicit weights that connect N features to n outputs. A kernel model uses fewer weights but the relationships between variables are stored implicitly. The dictionary-based kernel model selects the most active samples and therefore uses fewer weights still. (Online version in colour.)

We will show that it is possible to improve the scaling further by using a kernel defined on a sparse dictionary $\tilde{X}$. Figure 3 shows our dictionary-based kernel modelling procedure, where the explicit model on the left is a SINDy model, and the compact model on the right is our kernel model. Thus, our kernel learning approach may be viewed as a kernelized SINDy without sparsity promotion.

Based on the implicit LANDO model, it is possible to efficiently extract the linear component L of the dynamics, along with a matrix for the nonlinear forcing

2.7 Y=LX+N,

where here $N=\left[\begin{array}{cccc} N(x_{1}) & N(x_{2}) & \cdots & N(x_{m}) \end{array}\right]$ is a nonlinear snapshot matrix, where each column is the nonlinear component of the dynamics at that instant in time. Although this is not an explicit expression for the nonlinear dynamics, as in SINDy, knowing the linear model and nonlinear forcing will enable data-driven resolvent analysis [36], even for strongly nonlinear systems. Technically, (2.7) may be centred at any base point $\bar{x}$, resulting in

2.8 $$y_{j}^{\prime }=L^{\prime }x_{j}^{\prime }+N(x_{j}^{\prime }),$$

where $x^{\prime }=x-\bar{x}$. We will also show that the linear model L may be represented efficiently without being explicitly constructed, as in DMD.

Electronic supplementary material SI §C includes further comparison of LANDO to related data-driven architectures. The eDMD algorithm has already been kernelized [6,7], enabling efficient approximations to the Koopman operator with very large feature spaces. Although it is related to the present work, the goal of eDMD/kDMD is to obtain a *square* representation of the dynamics of measurement functions in a Hilbert space or feature space ϕ(x), rather than a closed representation of the dynamics in the original state x. In this way, our approach more closely resembles the SINDy procedure, but kernelized to scale to arbitrarily large problems. We will also show that even though the representation of the dynamics is implicit, it is possible to extract explicit model structures, such as the linear component and other relevant quantities, from the kernel representation.

In the following subsections, we will outline the DMD algorithm and provide an introduction to the kernel methods that will be used throughout this work.

### (a) Dynamic mode decomposition

The original DMD algorithm of [2] was developed as a data-driven method for decomposing high-dimensional snapshot data into a set of coherent spatial modes, along with a low-dimensional model for how these mode amplitudes evolve linearly in time. As such, DMD may be viewed as a hybrid algorithm combining PCA in space and the discrete-time Fourier transform in time [37]. DMD has been adopted in a wide range of fields beyond fluid mechanics, including epidemiology [38], neuroscience [39], video processing [40], robotics [41] and plasma physics [42]. Much of this success stems from the formulation of DMD as a linear regression problem [4], based entirely on measurement data, resulting in several powerful extensions [5], including for control [18], sparsity promoting DMD [43], for non-sequential time series [4,19] and for data that are under-resolved in space [44] or time [45].

The original algorithm was refined by [4] who phrased DMD in terms of the Moore–Penrose pseudoinverse thereby allowing snapshots that are not equally spaced in time; this variant is called *exact DMD* and will be the main form of DMD used in this paper. In the electronic supplementary material, appendix C(a), we will show that exact DMD may be viewed as a special case of our new method.

As mentioned in the previous section, it is assumed that each $x_{j}$ and $y_{j}$ are connected by a dynamical system of the form $y_{j}=F(x_{j})$. The aim of DMD is to learn information about F by approximating it as a linear operator and then performing diagnostics on that approximation. In particular, DMD seeks the linear operator A that best maps the sets $\left\{x_{j}\right\}$ and $\left\{y_{j}\right\}$ into one another:

2.9 $$y_{j}\approx Ax_{j}\;\text{for }j=1,\ldots ,m.$$

Expressed in terms of the snapshot matrices in (2.2), (2.9) becomes

2.10 Y≈AX,

and the minimum-norm solution is

2.11 $$A=\underset{A}{\mathrm{argmin}}||Y-AX|{|}_{F}=YX^{†},$$

where † indicates the Moore–Penrose pseudoinverse [46]. If X has the singular value decomposition (SVD) $X={U\Sigma V}^{*}$ then $A=YV\Sigma^{†}U^{*}$. Note that A is an n×n matrix so may be extremely large in practice where n≫1. Thus, it is common to use a rank-r approximation for A, denoted by $\hat{A}$, where r≪n. To construct $\hat{A}$, we build a rank r approximation for X using the truncated SVD: $X\approx U_{r}\Sigma_{r}V_{r}^{*}=X_{r}$. This approximation is optimal according to the Eckart–Young theorem [47]. The matrix A is then projected onto the column space of $X_{r}$ as

2.12 $$\hat{A}=U_{r}^{*}AU_{r}=U_{r}^{*}YV_{r}\Sigma_{r}^{-1}.$$

Since $\hat{A}$ is an r×r matrix, it is now feasible to compute its eigendecomposition as

2.13 $$\hat{A}\hat{\Psi }=\hat{\Psi }\Lambda .$$

It was proved by [4] that the eigenvectors of the full matrix A can be approximated from the reduced eigenvectors Ψ by

2.14 $$\Psi =YV\Sigma^{-1}\hat{\Psi }.$$

This eigendecomposition has many favourable properties. Firstly, it is an approximation to the spectrum of the underlying Koopman operator of the system [3]. Secondly, if the snapshots are equally spaced in time and $y_{j}=x_{j+1}$ then the data can be reconstructed in terms of the eigenvectors and eigenvalues as

2.15 $$x_{j}=\Psi \Lambda^{j-1}a,$$

where the vector a contains the mode amplitudes often computed as $a=\Psi^{†}x_{1}$. The above provides a clear physical interpretation of the modes: the eigenvectors Ψ are the spatial modes whereas the eigenvalues Λ correspond to the temporal evolution.

### (b) Kernel methods

Kernel methods are a class of statistical machine learning algorithms that perform efficient computations with high-dimensional nonlinear features [26]. Kernel methods have found applications in adaptive filtering [48], nonlinear principal component analysis [49], nonlinear regression [32], classification [50] and support vector machines [51]. The broad success of kernel machines stems from their ability to efficiently compute inner products in a high-dimensional, or even infinite-dimensional, nonlinear feature space. Thus, if a conventional linear algorithm can be phrased exclusively in terms of inner products then it can be ‘kernelized’ and adapted for nonlinear problems. This ‘kernel trick’ has been used to great effect in the above applications.

Kernels are continuous functions $k:R^{n}\times R^{n}\to R$, and a kernel is a Mercer kernel if it is positive definite; i.e. for any collection of vectors $x_{j}^{\prime }\in R^{n}$, the matrix K defined by ${\left[K\right]}_{i,j}=k(x_{i}^{\prime },x_{j}^{\prime })$ is positive definite. By Mercer’s theorem [52], it follows that there exists a Hilbert space $H_{k}$ and a mapping $\phi :R^{n}\to H_{k}$ such that $k(x,x^{\prime })=\left\langle \phi (x),\phi (x^{\prime })\right\rangle$. In other words, every Mercer kernel can be interpreted as an inner product in the Hilbert space $H_{k}$, which may be of an otherwise inaccessible dimension. Every element $g\in H_{k}$ can be expressed as a linear combination

2.16 $$g(x)=\sum_{j=1}^{M}\alpha_{j}k(x_{j}^{\prime },x),$$

for some M∈N, $\alpha_{i}\in R^{n}$ and $x_{j}^{\prime }\in R^{n}$. We drop the word ‘Mercer’ in the remainder of the article, and assume that all kernels are Mercer kernels.

An important result in the theory of kernel learning is the *representation theorem*. First proved by [53] and then generalized by [54], the representation theorem provides very general conditions where kernel methods can be used to solve machine learning problems. For the purposes of the present work, the representation theorem may be stated thus: for a set of pairs of m training samples, $\left(x_{1},y_{1}),\ldots ,(x_{m},y_{m}\right)$, the solution to the minimization problem

2.17 $$\underset{f\in H_{k}}{\mathrm{argmin}}||Y-f(X)|{|}_{F}+\lambda R(f),$$

may be expressed as

2.18 $$f(x)=\sum_{j=1}^{m}w_{j}k(x_{j},x),$$

for vectors $w_{j}\in R^{n}$. One important consequence of the representation theorem is that the solution to the optimization problem (2.17) can be expressed as a linear combination of kernel functions whose first arguments are the training data. Contrast this with the general representation of members of $H_{k}$ in (2.16) where the parameters $x_{j}^{\prime }$ are not known. The representation theorem allows us to avoid an exhaustive search for the optimal parameters, thereby reducing the problem to a (linear) search for the weights $w_{j}$. In the above, λ>0 is a regularization parameter and the regularizer on f is to be interpreted as the norm associated with k [27].

#### (i) An illustrative example

The discussion of kernels has thus far been rather abstract; we now make the theory concrete by illustrating an application of the usefulness of kernel methods. This simple example is often used in kernel tutorials [7,26].

Consider a three-dimensional state, $x\in R^{3}$, upon which we want to perform some machine learning task such as regression or classification. Suppose that we know—from either physical intuition, empirical data or experience—that the system is governed by pairwise quadratic interactions between the state variables. Thus, our machine learning model should operate in the nonlinear feature space defined by

2.19 $$\phi (x)={\left[x_{1}x_{2}\;x_{1}x_{3}\;x_{2}x_{3}\;x_{1}^{2}\;x_{2}^{2}\;x_{3}^{2}\right]}^{T}\in R^{6}.$$

Almost every machine learning algorithm uses inner products to measure correlations between samples. Computing inner products in a feature space of dimension N costs 2N−1 operations: N products and N−1 summations. Thus, in this example, computing inner products in the nonlinear feature space would usually require 11 operations. However, we still need to form the two feature vectors ϕ(x) and $\phi (x^{\prime })$, which cost a further six operations each, raising the total count to 23 operations.

Equivalently, we could build our model in the slightly rescaled feature space

2.20 $$\varphi (x)={\left[\sqrt{2}x_{1}x_{2}\;\sqrt{2}x_{1}x_{3}\;\sqrt{2}x_{2}x_{3}\;x_{1}^{2}\;x_{2}^{2}\;x_{3}^{2}\right]}^{T}.$$

Now note that inner products in this feature space may be expressed as

2.21 $$\begin{array}{rl} \left\langle \varphi (x),\varphi (x^{\prime })\right\rangle & \;=2x_{1}x_{1}^{\prime }x_{2}x_{2}^{\prime }+2x_{1}x_{1}^{\prime }x_{3}x_{3}^{\prime }+2x_{2}x_{2}^{\prime }x_{3}x_{3}^{\prime }+x_{1}^{2}{\left(x_{1}^{\prime }\right)}^{2}+x_{2}^{2}{\left(x_{2}^{\prime }\right)}^{2}+x_{3}^{2}{\left(x_{3}^{\prime }\right)}^{2}\\ & \;={\left(x_{1}x_{1}^{\prime }+x_{2}x_{2}^{\prime }+x_{3}x_{3}^{\prime }\right)}^{2}\\ & \;={\left(\left\langle x,x^{\prime }\right\rangle \right)}^{2}. \end{array}$$

Thus, we can compute the inner product $\left\langle \varphi (x),\varphi (x^{\prime })\right\rangle$ in merely six operations by computing the inner product $\left\langle x,x^{\prime }\right\rangle$ and then squaring the result. In other words, computing the inner product amounted to evaluating the kernel $k(u,v)={\left(u^{T}v\right)}^{2}$. Moreover, while computing the inner product with the kernel, we never explicitly formed the feature space, and therefore did not need to store φ(x) in memory. In summary, if we use expression (2.21) then the cost of computing inner products falls from 23 operations to six operations.

This may seem a modest saving but the cost of computations in feature space explodes as the state dimension or degree of nonlinearity increase. For a state of dimension n, the number of degree d monomial features is N=(n+d−1d)=(n+d−1)!/(d! (n−1)!).^1^ Thus, explicitly forming vectors in this feature space is extremely expensive, as is computing inner products. For example, for an n-dimensional state, the number of possible quadratic interactions between states is n(n−1)/2. This scaling of the feature vector is the prime limitation of SINDy.

Instead of explicitly forming this vast feature space, we instead work with suitably chosen kernels. The feature space of degree d monomials can be represented using the *polynomial kernel*

2.22 $$k(u,v)={\left(u^{T}v\right)}^{d}.$$

Thus, using the kernel (2.22) to compute inner products reduces the operation count from 2N−1 to 2n, which is significant when the state space is large and the nonlinearity is quadratic or higher.

## 3. Learning kernel models with sparse dictionaries

We now develop the main machine learning method presented by this paper. The procedure is based on the KRLS algorithm of [32] but is more stable and allows further interpretation and analysis of the learned model. We specifically tailor this approach to learn dynamical systems in a robust and interpretable framework. Recall that we are solving the optimization problem defined in (2.4) for a nonlinear function f that approximates the dynamics. By the representation theorem, we may express the dynamical system approximation f from (2.3) in the kernelized form (2.18) as

3.1 $$f(x)=\sum_{j=1}^{N}\xi_{j}\phi_{j}(x)=\sum_{j=1}^{m}w_{j}\left\langle \phi (x_{j}),\phi (x)\right\rangle =\sum_{j=1}^{m}w_{j}k(x_{j},x).$$

Arranging the column vectors $w_{j}$ into a matrix W allows us to write f(x)=W k(X,x) so the optimization problem is

3.2 $$\underset{W}{\mathrm{argmin}}||Y-Wk(X,X)|{|}_{F}+\lambda R(f).$$

Theoretically, a solution to (3.2), in the absence of regularization, is provided by the Moore–Penrose pseudoinverse:

3.3 $$W=Yk{\left(X,X\right)}^{†}.$$

As noted by [32], there are three practical problems with the above solution

Numerical conditioning: even though the kernel matrix may formally have full rank, it will usually have a very large condition number since the samples can be almost linearly dependent in the feature space. When the condition number is large, the condition number of the pseudoinverse will also be large and W will amplify noise by a corresponding amount.
Overfitting: the weight matrix W has mn entries, which is equal to the number of constraining equations in (3.2). Thus, there is a risk of overfitting, which can limit the generalizability of the model and make it sensitive to noise.
Computational complexity: in nonlinear system identification, we usually need a large number of samples to adequately learn the system. When there are m≫1 samples, constructing the pseudoinverse $k{\left(X,X\right)}^{†}$ requires $O(m^{3})$ operations to construct and $O(m^{2})$ space in memory, which can become prohibitively expensive. Additionally, evaluating the model f for prediction or reconstruction requires multiplying the n×m weight matrix by the m-vector of kernel evaluations, which will also become expensive for large sample sets.

To address these issues, Engel *et al.* [32] proposed an online form of dimensionality reduction that iteratively constructs a dictionary of samples that capture the salient features of the underlying dynamics. The key idea is that the model f defined in (2.18) can be approximated by

3.4 $$f(x)\approx \tilde{W}k(\tilde{X},x),$$

for a suitable choice of $\tilde{X}$ known as the *dictionary* (in this paper the tilde symbol indicates that a quantity is connected to the dictionary). Then, the optimization (3.2) may be approximated as

3.5 $$\underset{\tilde{W}}{\mathrm{argmin}}||Y-\tilde{W}k(\tilde{X},X)|{|}_{F}+\lambda R(f).$$

The dictionary is constructed by considering each sample and determining whether it should be included in the dictionary. Membership of a sample in the dictionary is decided by checking if the sample can be approximated in the feature space using the current dictionary. This scheme is called the ‘almost linearly dependent’ (ALD) test: if a sample is almost linearly dependent on the current dictionary then it is not added, otherwise the dictionary must be updated with the current sample. Thus, the dictionary is a sparse^2^ subset of samples that spans the largest subspace in the data. Usually, the size of the dictionary is much smaller than the number of samples. Physically, the selected samples are those that most substantially contribute to the dynamics, as measured by the kernel k.

The dictionary learning procedure searches the high-dimensional feature space for a low-dimensional subspace where most of the dynamics take place. This approach is similar to kernel principal component analysis (KPCA [49]), though we argue that ALD dictionary learning is more physically interpretable. KPCA conflates the feature space representations of samples, and the result usually has no interpretation in the original physical space. For example, if the feature space is $\phi (x)={\left[x_{1}\;x_{2}\;x_{1}x_{2}\right]}^{T}$ then certain datasets could produce a principal component of $\hat{\phi }={\left[1\;1\;0\right]}^{T}$. However, such a vector is unrealizable in the original physical space because if the $x_{1}x_{2}$ component is zero then at least one of $x_{1}$ and $x_{2}$ must also be zero. It was shown in [32] that ALD dictionary learning may be viewed as an approximate form of KPCA. Additionally, the dictionary has a clear physical interpretation since every member it contains is simply the state vector system at a specific time. Thus, ALD dictionary learning may be preferable to KPCA when studying physically motivated problems.

### (a) Sparse dictionary learning

The dictionary at time t is defined as a collection of ${\tilde{m}}_{t}$ vectors, $D_{t}=\{{\tilde{x}}_{j}|j=1,\ldots ,{\tilde{m}}_{t}\}$, and is initialized with $D_{1}=\{x_{1}\}$. We write

3.6 $$X_{t}=\left[\begin{array}{cccc} | & | & & |\\ x_{1} & x_{2} & \cdots & x_{t}\\ | & | & & | \end{array}\right],$$

to represent the data matrix including all samples up to snapshot t. We may also represent the dictionary in terms of data matrices in the state space and feature space, respectively, as

3.7 $${\tilde{X}}_{t}=\left[\begin{array}{cccc} | & | & & |\\ {\tilde{x}}_{1} & {\tilde{x}}_{2} & \cdots & {\tilde{x}}_{{\tilde{m}}_{t}}\\ | & | & & | \end{array}\right]\;\mathrm{and}\;{\tilde{\Phi }}_{t}=\left[\begin{array}{cccc} | & | & & |\\ \phi ({\tilde{x}}_{1}) & \phi ({\tilde{x}}_{2}) & \cdots & \phi ({\tilde{x}}_{{\tilde{m}}_{t}})\\ | & | & & | \end{array}\right].$$

When a new element is introduced, we determine how much new information it could add to our model. In other words, how well can the new element be approximated using the members of the current dictionary. The degree to which the current sample can be well represented by the dictionary in feature space is quantified by

3.8 $$\delta_{t}=\min_{\pi_{t}}||\phi (x_{t})-{\tilde{\Phi }}_{t-1}\pi_{t}|{|}_{2}^{2}.$$

The number $\delta_{t}$ represents the minimum (squared) distance between the current sample and the span of the current dictionary and $\pi_{t}$ specifies the linear combination of dictionary elements that minimizes this distance. Having calculated $\delta_{t}$ as detailed below, we compare it with a user-defined sparsification threshold ν. If $\delta_{t}\leq \nu$ then the new sample $x_{t}$ can be approximated in the feature space using linear combinations of members of the dictionary. Thus, the new sample is ALD on the dictionary elements in the implicit feature space. If $\delta_{t}>\nu$ then the new sample cannot be well approximated by the current dictionary. Thus, the new sample contributes meaningful information that was not already present in the dictionary and the dictionary should be updated with the current sample.

By expanding the norm in (3.8) and using properties of kernels, we can show that

3.9 $$\delta_{t}=k_{tt}-{\tilde{k}}_{t-1}^{*}\pi_{t},$$

where the minimizer is

3.10 $$\pi_{t}={\tilde{K}}_{t-1}^{-1}{\tilde{k}}_{t-1}$$

and

3.11 $$k_{tt}=k(x_{t},x_{t})\;\mathrm{and}\;{\tilde{k}}_{t-1}=k({\tilde{X}}_{t-1},x_{t})\in R^{{\tilde{m}}_{t-1}}.$$

The kernel matrix ${\tilde{K}}_{t-1}^{-1}$ and its inverse should be updated whenever an element is added to the dictionary. The updated equations are, respectively,

3.12 $${\tilde{K}}_{t}=\left[\begin{array}{cc} {\tilde{K}}_{t-1} & {\tilde{k}}_{t-1}\\ {\tilde{k}}_{t-1}^{*} & k_{tt} \end{array}\right]\;\mathrm{and}\;{\tilde{K}}_{t}^{-1}=\left[\begin{array}{cc} {\tilde{K}}_{t-1}^{-1}+\frac{\pi_{t}\pi_{t}^{*}}{\delta_{t}} & \frac{-\pi_{t}}{\delta_{t}}\\ \frac{-\pi_{t}^{*}}{\delta_{t}} & \frac{1}{\delta_{t}} \end{array}\right].$$

The above expression for ${\tilde{K}}_{t}^{-1}$ is mathematically correct but numerically unstable. This issue is typical of kernel methods, which are often plagued with problems of numerical stability due to the large condition numbers associated with kernel matrices. This seems to not be an issue for the Gaussian kernels that were used in the original KRLS formulation of [32], but it becomes important when working with the polynomial kernels that arise in physical applications. To circumvent these issues, we avoid constructing the ill-conditioned matrices ${\tilde{K}}_{t}$ and ${\tilde{K}}_{t}^{-1}$ explicitly. In particular, as ${\tilde{K}}_{t}$ is positive definite it admits a unique Cholesky decomposition ${\tilde{K}}_{t}=C_{t}C_{t}^{*}$ where $C_{t}$ is a lower-triangular ${\tilde{m}}_{t}\times {\tilde{m}}_{t}$ matrix. Instead of updating ${\tilde{K}}_{t}$ according to (3.12), we instead update and store its Cholesky factor. The Cholesky factor is initialized as $C_{1}=\sqrt{k_{11}}$ and the updated rule is

3.13 $$C_{t}=\left[\begin{array}{cc} C_{t-1} & 0\\ s_{t}^{*} & c_{t} \end{array}\right],$$

where $s_{t}=C_{t-1}^{-1}{\tilde{k}}_{t-1}$ can be formed in $O({\tilde{m}}_{t}^{2})$ operations by backsubstitution and $c_{t}=\sqrt{k_{tt}-||s_{t}|{|}_{2}^{2}}$. Rounding errors can still accumulate and produce an imaginary value for $c_{t}$, so in practice one can use $c_{t}=\max(0,\sqrt{k_{tt}-||s_{t}|{|}_{2}^{2}})$. In summary, multiplication by the inverse ${\tilde{K}}_{t}^{-1}$ should be interpreted and implemented as solving a linear system with two back substitutions of $C_{t}$. Thus, we can compute the distance of a sample from the dictionary (3.9) without ever forming the ill-conditioned matrices ${\tilde{K}}_{t}$ and ${\tilde{K}}_{t}^{-1}$. The full dictionary can be learned in $O(m{\tilde{m}}^{2}+nm\tilde{m})$ time. Note that $s_{t}$ is formed implicitly in (3.10) so need not be computed explicitly. Before learning the dictionary, we strongly recommend that the order of samples in X is randomly permuted. This step avoids the situation where the first few samples are almost linearly independent, which can lead to dictionaries with large condition numbers.

**Figure 4.**
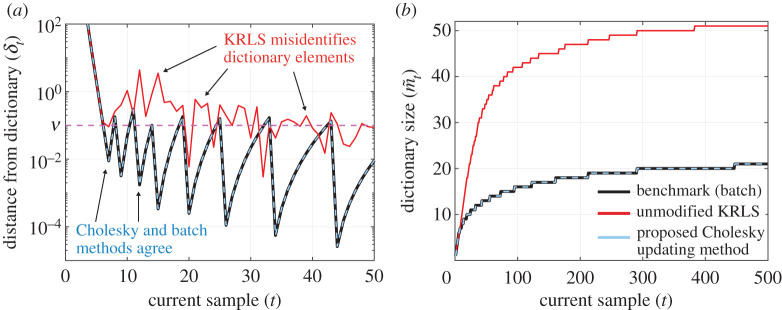
Comparing the ALD dictionaries computed by the original KRLS algorithm, the Cholesky updating variant and a batch offline algorithm when applied to a solution of the viscous Burgers’ equation. The kernel here is quadratic and the sparsity parameter is ν=0.1. (*a*) The computed distance of each sample from the span of the current dictionary, which determines whether the current sample should be added to the dictionary. (*b*) Plots the growth of the dictionary as more samples are considered. The original KRLS algorithm misidentifies dictionary elements and the corresponding dictionary is larger than necessary. (Online version in colour.)

Updated Cholesky factors significantly improve dictionary learning. Figure 4 illustrates the improvement by comparing three methods of dictionary learning. We evaluate the efficacy of each method by their accuracy in computing $\delta_{t}$ for each sample, which represent the algorithm’s estimate of the distance of sample t from the current dictionary. Recall that $\delta_{t}$ determines whether the current sample should be included in the dictionary so accurate computation of $\delta_{t}$ is essential. The first method is the original KRLS formulation, which uses (3.12) to compute $\delta_{t}$ with (3.10). The second method is the Cholesky updating formulation presented here, which uses (3.13) to compute $\delta_{t}$ as opposed to constructing ${\tilde{K}}_{t}$ or ${\tilde{K}}_{t}^{-1}$ explicitly. The third method is a batch method that computes ${\tilde{K}}_{t}^{-1}$ from scratch at each iteration and does not use the estimates of ${\tilde{K}}_{t-1}^{-1}$ or $C_{t-1}$ at the previous iteration. We take the batch method to be the ground truth, although there will still be some numerical instability associated with the large condition number of ${\tilde{K}}_{t}$. Although accurate, the batch method is also prohibitively expensive at large scales, with each iteration costing $O({\tilde{m}}^{3})$ as opposed to the updating methods, which cost merely $O({\tilde{m}}^{2})$. The data here are chronologically ordered samples from a simulation of the viscous Burgers’ equation (see §5c), and we use a quadratic kernel with sparsity threshold ν=0.1. Figure 4*a* indicates that the first seven samples are all added to the dictionaries. After this transient period, most new samples are well represented by the current dictionary and are therefore excluded. Occasionally, the data drift sufficiently far from the dictionary that a new sample must be included. This is illustrated by the spikes appearing in the batch method and the Cholesky updating method in figure 4*a*. Physically, this indicates that the solution of the PDE has departed from what can be adequately described by the dictionary of previous samples. The results indicate that the batch method and Cholesky updating method select identical dictionaries, whereas the KRLS dictionary learning algorithm misidentifies a large number of dictionary elements. Moreover, figure 4*b* shows that the KRLS dictionary is more than twice the size of the correct dictionary. In summary, figure 4 indicates that the Cholesky factor method significantly improves the accuracy of the learned dictionaries.

Pseudocode for the dictionary learning procedure may be found in the electronic supplementary material, SI §A.

### (b) Batch regression learning

Once the dictionary has been learned, the optimization becomes the tractable problem defined in (3.5). There are many methods available to find the weights $\tilde{W}$ from (3.5); in the absence of further regularization on $\tilde{W}$, we use the Moore–Penrose pseudoinverse

3.14 $$\tilde{W}=Y\;k{\left(\tilde{X},X\right)}^{†}.$$

The computation of the pseudoinverse is far cheaper than the full solution (3.3) and avoids the issues described earlier. Thus, we are left with two quantities that together define the nonlinear model in (3.4): the final dictionary matrix $\tilde{X}$ with $\tilde{m}$ columns and the final set of weights $\tilde{W}$.

The model may also be learned in a purely online fashion (see the electronic supplementary material, appendix D), which is useful when working with streaming data. The algorithm is also applicable to situations where the system is forced by an exogenous control variable: details are provided in the electronic supplementary material, appendix E.

## 4. Extracting and enforcing physical structure with kernel machines

Having calculated the kernel weights $\tilde{W}$, we may now construct our model $f(x)=\tilde{W}\;k(\tilde{X},x)$ from (3.4). This kernel model is *implicit*: without further analysis we cannot interpret the model and understand the physical relationships that the model has learned. In this section, we present techniques that extract physically interpretable structures from the kernel model f.

### (a) Extracting structure from kernel machines: the linear operator

One means of providing insight and interpretability is to analyse the linear component of f relative to some state. In particular, suppose we consider perturbations (not necessarily of small amplitude) about a *base state* $\bar{x}$ which may correspond to the mean of the data, an equilibrium solution, or simply the zero vector. We define the perturbations about the base state as $x^{\prime }$ so that $x=\bar{x}+x^{\prime }$. A typical approach is to seek a representation of our model of the form

4.1 $$f(x)=c+Lx^{\prime }+N(x^{\prime }),$$

where L is a linear transformation, c is a constant and N is a nonlinear operator such that

4.2 $$\lim_{||x^{\prime }|{|}_{2}\to 0}\frac{||N(x^{\prime })|{|}_{2}}{||x^{\prime }|{|}_{2}}=0.$$

In words, condition (4.2) restricts N so that it is purely nonlinear with respect to the base state $\bar{x}$. If L and c are known, then rearranging (4.1) obtains the nonlinear fluctuations as $N(x^{\prime })=f(x)-L(x^{\prime })-c$.

Numerically computing the linear component of a high-dimensional nonlinear operator can be computationally expensive. For example, neural networks use stochastic gradient descent to estimate the local slopes of high-dimensional functions for optimization. By virtue of our use of kernels, we can extract the linear component analytically.

We consider the Taylor expansion of f about $\bar{x}$

4.3 $$f(x)=f(\bar{x})+\nabla f(x){|}_{x=\bar{x}}x^{\prime }+\text{higher-order terms}.$$

Thus, f(x) can be expressed in the form (4.1) where

$$c=f(\bar{x}),\;L=\nabla f(x){|}_{x=\bar{x}}\;\mathrm{and}\;N(x^{\prime })=f(x)-f(\bar{x})-\nabla f(x){|}_{x=\bar{x}}x^{\prime }.$$

Accordingly, to compute c, L and N, we need only compute f and ∇f. Since our model consists of linear combinations of kernels (3.4), the gradient is simply

4.4 $$\nabla f(x){|}_{x=\bar{x}}=\tilde{W}\nabla k(\tilde{X},\bar{x}).$$

Evidently, the linearization depends on the choice of kernel, so the kernel should be carefully designed with this in mind (§4d). The gradients ∇k can usually be computed analytically in a straightforward manner. For example, for polynomial kernels (see (4.20) and §4d), we have

4.5 $$\nabla k(\tilde{X},\bar{x})=\text{diag}[d{\left(c+{\tilde{x}}_{j}^{T}\bar{x}\right)}^{d-1}]{\tilde{X}}^{*}.$$

For Gaussian kernels (see (4.21)), the gradient is

4.6 $$\nabla k(\tilde{X},\bar{x})=\text{diag}\left[\frac{-1}{\sigma^{2}}\exp\left(\frac{-||{\tilde{x}}_{j}-\bar{x}|{|}_{2}^{2}}{2\sigma^{2}}\right)\right]{\left(\tilde{X}-\bar{X}\right)}^{*},$$

where $\bar{X}$ is an $n\times \tilde{m}$ matrix where each column is $\bar{x}$. Similar expressions can be derived for any kernel function or any combination of kernels.

Note that the gradients (4.5) and (4.6) all take the form $\nabla k=S{\left(\tilde{X}-\rho \bar{X}\right)}^{*}$, where S is a diagonal matrix and either ρ=0 or ρ=1. Indeed, an application of the chain rule shows that ∇k takes this form for any distance kernel (ρ=1) or inner product kernel (ρ=0) as defined in §4d. Thus, for these extremely broad classes of kernels, the linear operator may be expressed in the general form

4.7 $$L=\tilde{W}S{\left(\tilde{X}-\rho \bar{X}\right)}^{*}.$$

In the case $\tilde{m}\ll n$, the expression (4.7) is computationally attractive since L need not be stored explicitly; instead of storing a large n×n matrix, it is sufficient to store two $n\times \tilde{m}$ matrices and a diagonal $\tilde{m}\times \tilde{m}$ matrix. Additionally, the potentially expensive matrix multiplications involved in forming L can be avoided. For example, it is not necessary to form L explicitly if all that is required is its eigendecomposition, as with DMD.

### (b) Extracting structure from kernel machines: the dynamic mode decomposition

We can exploit the factorization in (4.7) to perform a DMD of the linear operator L. This step can be computationally expensive as L is an n×n matrix so the eigendecomposition costs $O(n^{3})$ operations. However, we can obtain the leading eigenvectors and eigenvalues by computing the eigendecomposition of a much smaller matrix that is (at most) $\tilde{m}\times \tilde{m}$. This idea is formalized in the following lemma.

**Lemma 4.1. (Dynamic mode decomposition of the linear operator)**

*Let* $(\tilde{X}-\rho \bar{X})S=U\Sigma V^{*}$ *be the (economy) SVD of the rescaled and shifted dictionary and* $\hat{L}=U^{*}LU$ *be the projection of* L *from* (4.7) *onto the columns of* U. *If* $\hat{L}\hat{\psi }=\lambda \hat{\psi }$ *with* λ≠0 *then*

4.8 $$\psi =\frac{1}{\lambda }\tilde{W}V\Sigma \hat{\psi },$$

*is an eigenvector of* L *with eigenvalue* λ. *Additionally, all non-zero eigenvalues of* L *are eigenvalues of* $\hat{L}$.

The operator $\hat{L}$ represents the projection of the full linear operator L onto the principal components (proper orthogonal decomposition modes) of the rescaled and shifted dictionary $(\tilde{X}-\rho \bar{X})S$. This lemma is significant since it implies that every non-zero eigenvalue of L can be obtained by computing the eigendecomposition of the smaller matrix $\hat{L}$. Furthermore, the eigendecomposition produces an eigenvector of L that corresponds to each eigenvalue.

We now prove the lemma using similar arguments to those used in theorem 1 of [4].

*Proof*. We first show that the pair (ψ,λ) is indeed an eigenvector/eigenvalue pair. Assume that $\hat{L}\hat{\psi }=\lambda \hat{\psi }$ for λ≠0 and define

4.9 $$G=\tilde{W}V\Sigma ,$$

so that $\psi =(1/\lambda )G\hat{\psi }$. By (4.7) and the economy SVD, we may write L as

4.10 $$L=\tilde{W}{\left(U\Sigma V^{*}\right)}^{*}=\tilde{W}V\Sigma U^{*}=GU^{*}.$$

Similarly,

4.11 $$\hat{L}=U^{*}(\tilde{W}{\left(U\Sigma V^{*}\right)}^{*})U=U^{*}\tilde{W}V\Sigma =U^{*}G.$$

Thus,

4.12 $$L\psi =\frac{1}{\lambda }(GU^{*})(G\hat{\psi })=\frac{1}{\lambda }G\hat{L}\hat{\psi }=G\hat{\psi }=\lambda \psi$$

as required.

We will now prove that every non-zero eigenvalue of L is also an eigenvalue of $\hat{L}$. Let (ψ,λ) be a non-zero eigenvector/eigenvalue pair and define $u=U^{*}\psi$. Then

4.13 $$\hat{L}u=U^{*}GU^{*}\psi =U^{*}L\psi =\lambda U^{*}\psi =\lambda u.$$

Note also that u is not the zero vector. If it were, then $L\psi =GU^{*}\psi =Gu=0$ and therefore λ=0, which contradicts our assumption that λ≠0. Combining this observation with (4.13) shows that λ is also an eigenvalue of $\hat{L}$.

Pseudocode for extracting the linear operator and computing the DMD is available in the electronic supplementary material, SI §A. In the electronic supplementary material, SI §C, we demonstrate that we recover the exact DMD formulation [4] in the special case of a linear kernel.

### (c) Extracting structure from kernel machines: querying nonlinear relationships

The analysis of §4a showed that we can extract linear relationships from otherwise opaque kernel machines. This section demonstrates that we can also extract specific nonlinear relationships between the input and output states.

Suppose that we know that the implicit feature space consists of a specific nonlinear scalar feature of interest labelled $\phi_{j}(x)$. For example, we may be interested in the effect of quadratic interactions between two states: $\phi_{j}(x)=x_{1}x_{2}$. To ‘query’ $\phi_{j}(x)$ is to determine the n-dimensional vector that represents the effect of the nonlinearity $\phi_{j}(x)$ on the elements of the output vector f(x). Without loss of generality, we can decompose the implicit feature vector ϕ(x) into $\phi_{j}(x)$ and $\phi^{\prime }(x)$, where $\phi^{\prime }(x)$ is the original feature vector with the $\phi_{j}(x)$ element removed. Applying (3.1) allows us to write

$$y=f(x)=\tilde{W}({\left(\phi_{j}(\tilde{X})\right)}^{*}\phi_{j}(x)+{\left(\phi^{\prime }(\tilde{X})\right)}^{*}\phi^{\prime }(x)).$$

Thus, the effect of the features $\phi_{j}(x)$ on f(x) can be determined by simply reading off its coefficient as $\tilde{W}{\left(\phi_{j}(\tilde{X})\right)}^{*}$. The result corresponds to the jth column of the explicit Ξ matrix of coefficients from (2.3).

### (d) Using partial knowledge of system physics to design kernels

An informed choice of kernel is critical to the success of kernel machines. Prior knowledge about the physical properties of a system can and should be considered when designing the kernel used for learning. This physical knowledge may include specific symmetries, invariants and conservation laws that are known to exist in the system under consideration. Moreover, in addition to enforcing known physics, it is possible to uncover these physical properties when they are unknown based on which kernel functions provide the best validated performance. In the next section, we will also see that it is possible to test several kernels, and by choosing the kernel with the best validated performance gain insight into what terms might be present in the governing equations.

The choice of kernel has many different perspectives as outlined in ch. 13 of [26]; the most useful perspective in this work is that the kernel defines the function space used by our model. For example, the kernel chosen in §2b(i) corresponded to the function space of quadratic monomials. Thus, that kernel can be used to model systems that are dominated by quadratic interactions between the states.

There are several strategies that can be used to design suitable kernels for a given physical problem. Useful references are ch. 13 of [26] and ch. 3 of [27]. Kernels can be combined to obtain new kernels, which affords significant flexibility when constructing kernels for a given problem. For example, the set of (Mercer) kernels forms a convex cone: for kernels $k_{1}$ and $k_{2}$, the conical combination

4.14 $$k(u,v)=\alpha_{1}^{2}k_{1}(u,v)+\alpha_{2}^{2}k_{2}(u,v),$$

is also a kernel. When two kernels are combined in this way, their feature space representations are scaled and stacked. If the kernels $k_{1,2}$ induce features $\phi_{1,2}$ then the features induced by k in (4.14) is $\left[\begin{array}{c} \alpha_{1}\phi_{1}\\ \alpha_{2}\phi_{2} \end{array}\right]$. This construction is useful when designing kernels for a given physical problem. For example, we may know that a system is dominated by linear and cubic interactions between its states. Thus, we may propose a kernel consisting of conical combinations of appropriate monomial kernels

4.15 $$k(u,v)=\alpha_{1}^{2}u^{T}v+\alpha_{3}^{2}{\left(u^{T}v\right)}^{3}.$$

This kernel induces a feature space consisting of purely linear and cubic terms

4.16 $$\phi (x)={\left[\begin{array}{cccccccc} \alpha_{1}x_{1} & \cdots & \alpha_{1}x_{n} & \alpha_{3}x_{1}^{3} & \sqrt{3}\alpha_{3}x_{1}^{2}x_{2} & \cdots & \sqrt{6}\alpha_{3}x_{n-1}x_{n-2}x_{n-3} & \alpha_{3}x_{n}^{3} \end{array}\right]}^{T}.$$

The constants $\alpha_{1,3}$ represent the relative importance of the linear and cubic terms and can be chosen through physical intuition or cross-validation.

Another useful result is that kernels are closed under direct sums. If $k_{1}:X_{1}\times X_{1}\to R$ and $k_{2}:X_{2}\times X_{2}\to R$ are kernels then their direct sum

4.17 $$\left(k_{1}⊕k_{2})(u,u^{\prime },v,v^{\prime })=k_{1}(u,v)+k_{2}(u^{\prime },v^{\prime }\right)$$

is a kernel on $\left(X_{1}\times X_{2})\times (X_{1}\times X_{2}\right)$. This fact can be exploited to design kernels where the inputs have different meanings or known physics implies different governing laws for the different states. For example, we could have a state space consisting of two types of measurements so $x=\left[\begin{array}{c} x^{\left(1\right)}\\ x^{\left(2\right)} \end{array}\right]$ where $x^{\left(j\right)}$ are $n^{\left(j\right)}$-dimensional vectors. Suppose also that it is known that the system is governed by a linear response to $x^{\left(1\right)}$ and quadratic interactions of $x^{\left(2\right)}$. An appropriate kernel for our model would then be

4.18 $$k(u,v)=\alpha_{1}^{2}(u^{(1)T}v^{\left(1\right)})+\alpha_{2}^{2}{\left(u^{(2)T}v^{\left(2\right)}\right)}^{2},$$

which induces the feature space

4.19 $$\phi (x)={\left[\alpha_{1}x_{1}^{\left(1\right)}\;\cdots \;\alpha_{1}x_{n^{\left(1\right)}}^{\left(1\right)}\;\alpha_{2}{\left(x_{1}^{\left(2\right)}\right)}^{2}\;\sqrt{2}\alpha_{2}x_{1}^{\left(2\right)}x_{2}^{\left(2\right)}\;\cdots \;\alpha_{2}{\left(x_{n^{\left(2\right)}}^{\left(2\right)}\right)}^{2}\right]}^{T}.$$

Thus far we have only explained how to design kernels for purely polynomial models. Non-polynomial terms play an important role in many nonlinear systems [55], and these can easily be incorporated into kernel design. For example, l may represent a vector of pointwise trigonometric functions that we wish to incorporate into our feature space. The corresponding kernel is simply $k(u,v)=l{\left(u\right)}^{T}l(v)$, which can be combined with any other kernel to supplement the feature space with the non-polynomial nonlinearities l.

Two classes of kernels have received significant attention in applications. Inner product kernels take the form $k(u,v)=\kappa (u^{T}v),$ where κ is a scalar function. Inhomogeneous polynomial kernels are inner product kernels that take the form

4.20 $$k(u,v)={\left(c+u^{T}v\right)}^{d},$$

where c is a constant and d∈N is the degree of polynomial. These inhomogeneous polynomial kernels are linear combinations of the monomial kernels in (2.22). The special case c=0 and d=1 corresponds to a linear feature space.

Distance kernels are another important class of kernels and take the form $k(u,v)=\kappa (||u-v|{|}_{2})$. A popular example used in several applications is the Gaussian kernel

4.21 $$k(u,v)=\exp\left(\frac{-||u-v|{|}_{2}^{2}}{2\sigma^{2}}\right),$$

where σ is a constant.

Supplementing the feature space with a bias term can be achieved by combining a kernel with a constant, such as $k=\alpha_{0}^{2}+\alpha_{1}^{2}k_{1}$, so that the feature space becomes $\left[\begin{array}{c} \alpha_{0}\\ \alpha_{1}\phi_{1} \end{array}\right]$. Additionally, pointwise products of kernels ($k=k_{1}k_{2}$) are also kernels and the corresponding features are the products of all pairs of features from the first and second feature space.

Kernels can also be designed to respect known physical invariances or symmetries [56]. For example, Klus *et al.* [57] recently derived analogues of the Gaussian and polynomial kernels that respect the symmetries of quantum physics. Models that respect such invariances and symmetries are highly desirable as they usually require less training data and are less prone to overfitting. Techniques for incorporating invariances into kernel machines are available in ch. 11 of [26].

To summarize this section, we have demonstrated that

(i) kernels can efficiently compute dense polynomial interactions between states that would otherwise be combinatorially complex;
(ii) kernels can be combined to generate a range of feature spaces;
(iii) if the features have different physical meanings or governing laws then one can construct separate models and combine the kernels using a direct sum;
(iv) non-polynomial and constant terms can be incorporated into kernels; and
(v) kernels can be designed to respect symmetries and invariances.

These observations indicate that there is significant flexibility for incorporating partially known physics into our models through a suitable choice of kernel. Similarly, the validated performance of a handful of candidate kernels may provide insight into underlying physics, such as symmetries and terms in the governing equations.

## 5. Results

**Figure 5.**
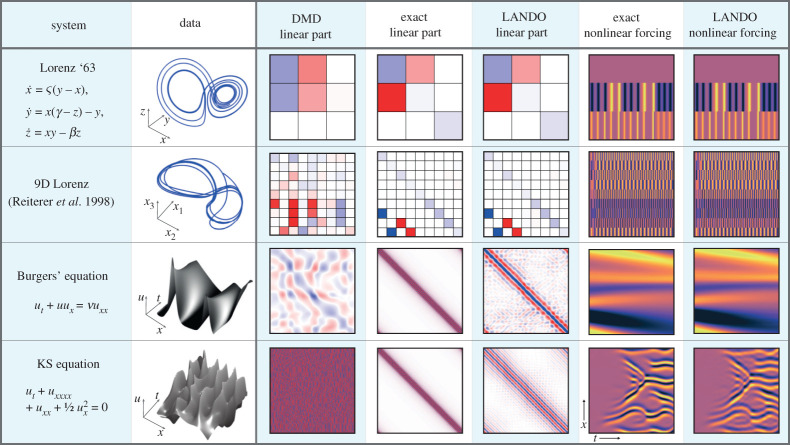
A comparison of learned linear operators for dynamical systems and PDEs. In the linear operators, red represents positive quantities whereas blue represents negative quantities. The problem sizes are provided in §5. (Online version in colour.)

We now demonstrate our approach on a range of physically relevant systems. We will consider both dynamical systems and high-dimensional discretized PDEs. The results of LANDO applied to these systems is summarized in figure 5. It can be seen that the true linear and nonlinear forcing components are accurately recovered by LANDO, while DMD fails to identify the correct linear model. For the dynamical systems considered, the linear operators are known exactly; for the PDEs, we express the ‘true’ linear operators as the appropriate spectral differentiation matrices. In addition to the Lorenz system (§5a), the viscous Burgers’ equation (§5c) and the KS equation (§5d), we also consider a 9D analogue of the Lorenz system [58]. Reiterer *et al.* derived this analogue by modelling dissipative Rayleigh–Benard convection in a three-dimensional cell and applying a triple Fourier expansion to the associated Bousinnesq–Oberbeck equations. The resulting analogue exhibits similar asymptotic behaviour to the original Lorenz system, including a low-dimensional chaotic attractor and a period-doubling cascade. We do not repeat the equations here for the sake of brevity, but they are analogous to the original Lorenz system and can be found in equation 18 of [58]. In particular, the equations consist of a linear operator and quadratic interactions between the states. We observe in figure 5 that we recover the linear component of the operator to a high degree of accuracy.

### (a) Implicit learning of the chaotic Lorenz system

We first illustrate our approach on the Lorenz system [59], which is a prototypical example of chaos and is often used in demonstrations of nonlinear system identification [8]

5.1 $$\dot{x}=\varsigma (y-x),\;\dot{y}=x(\gamma -z)-y\;\mathrm{and}\;\dot{z}=xy-\beta z,$$

where ς, β and γ are constants that parametrize the system. Our goal here is to use the LANDO framework to find data-driven local linearizations of (5.1). We are also interested in the predictive abilities of the full nonlinear model (3.4) learned by LANDO. Both the state dimension of the system (n=3) and the order of polynomial nonlinearity (d=2) are relatively small, so the benefits of kernel methods here are limited. As such, the system is considered here only for demonstration.

We take the standard parameter values ς=10, γ=28 and β=8/3 and initial condition x=−8, y=8 and z=27. The system (5.1) is integrated from t=0 to t=10 and the solution is sampled at time intervals of $\Delta t=10^{-3}$ resulting in 10 000 samples. The data matrix X comprises snapshots of the solution at each time step so that $x_{j}={\left[x(j\Delta t)\;y(j\Delta t)\;z(j\Delta t)\right]}^{T}$, and the columns of Y are the derivatives at each time: $y_{j}={\dot{x}}_{j}$. The order of the samples is randomly permuted so that the sparse dictionary is as rich as possible. The data used in this example are free of noise, and we demonstrate that the algorithm can be made robust to noise in the electronic supplementary material, appendix F.

**Figure 6.**
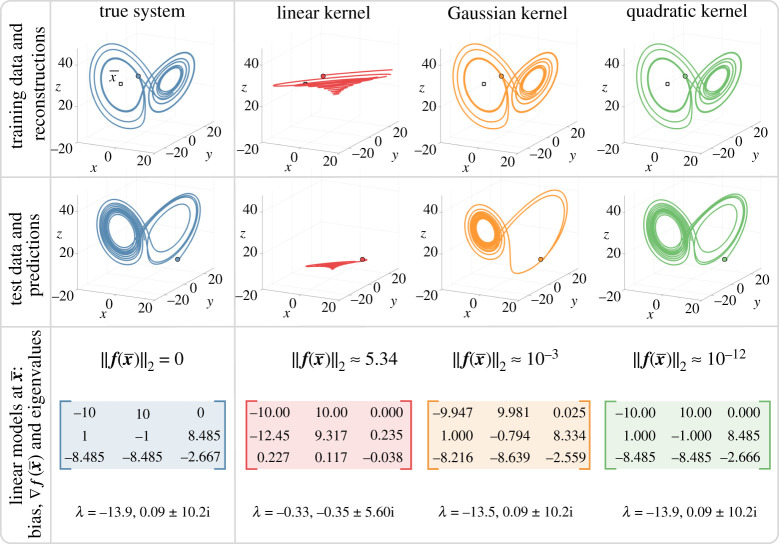
Kernel learning of the Lorenz system. We compare the learned models and predicted trajectories for linear, quadratic and Gaussian kernels. The training data are discrete-time snapshots of the state ${\left[x\;y\;z\right]}^{T}$ and the corresponding velocity measurements. The top row shows the models’ reconstructions of the training data, the middle row shows the predicted trajectory from a different initial conditions, and the bottom row shows the learned linear model near the equilibrium point $\bar{x}={\left[-\sqrt{\beta (\gamma -1)}\;-\sqrt{\beta (\gamma -1)}\;\gamma -1\right]}^{T}$, which is indicated by square. The parameter values are ς=10, γ=28 and β=8/3 and the initial conditions are represented by circle. (Online version in colour.)

The results of our kernel learning algorithm are illustrated in figure 6. We use LANDO to calculate four quantities: a reconstruction of the dynamics, a prediction of the dynamics for a different initial condition, the model error at a known equilibrium point and a local linear model at that equilibrium point. For each such quantity, we consider three types of kernels: linear ($k(u,v)=u^{T}v$), quadratic ($k(u,v)={\left(1+u^{T}v\right)}^{2}$) and Gaussian ($k(u,v)=\exp(-||u-v|{|}_{2}^{2}/(2\sigma^{2}))$ with σ=1.1). These kernels are not optimized, and the best kernel parameters may be chosen through cross-validation. The top row of figure 6 illustrates the reconstructions achieved by each model on the same initial condition used for training. Each reconstruction is created by integrating the learned kernel model $\dot{x}=f(x)$. The linear model performs poorly and reconstructs a decaying spiral. By contrast, the quadratic and Gaussian models accurately capture the behaviour of the underlying system. The quadratic model has a training error of $O(10^{-12})$. Higher-order polynomial kernels produce similar training errors to the quadratic model.

This example also illustrates the value of a sparse dictionary: applying a standard kernel regression to this problem would require inverting a large 10 000×10 000 matrix. Instead, the dictionary sizes are $\tilde{m}=3,7$ and 84 for the linear, quadratic and Gaussian kernels, respectively.

We also present the trajectories predicted by our models with the different initial condition of x=10, y=14 and z=10. The linear model prediction is poor and decays to the origin, while the Gaussian kernel model reproduces a trajectory that is roughly similar to the Lorenz system. Finally, as expected, the quadratic kernel model generates an excellent prediction, which indicates the generalizability of the model to trajectories away from the initial data.

We now extract meaningful physical information from the kernel models. Specifically, we extract linear models near the equilibrium $\bar{x}={\left[-\sqrt{\beta (\gamma -1)}\;-\sqrt{\beta (\gamma -1)}\;\gamma -1\right]}^{T}$, indicated by a square in the top row of figure 6. Note that $\bar{x}$ is not included in the training data, and different equilibrium points will produce different models and eigenvalues. Nevertheless, the quadratic and Gaussian models both identify $\bar{x}$ as an equilibrium since $||f(\bar{x})||$ is close to zero for both models. Moreover, applying the results of §4a to each model generates local linear models for the behaviour near $\bar{x}$. The true linearized model and the learned local linear models are reported in the third row of figure 6. All models capture the first row of the linearization, where the true system is also linear. However, the linear kernel model fails to estimate the rest of the linearization, while the quadratic and Gaussian kernel models provide excellent agreement; the local linear model learned by the quadratic kernel is correct to $O(10^{-4})$.

### (b) Extracting natural frequencies from densely coupled oscillators

We now use our framework to study systems of coupled oscillators from a data-driven perspective. The Kuramoto model is a prototypical model of coupling and synchronization, and has been applied to biological, chemical, physical and social systems [60]. We consider a forced Kuramoto model of n coupled oscillators of the form

5.2 $${\dot{\vartheta }}_{i}=\omega_{i}+\frac{1}{n}\sum_{j=1}^{n}a_{ij}\sin(\vartheta_{j}-\vartheta_{i})+h\sin(\vartheta_{j}),\;i=1,\ldots ,n,$$

where $\left\{\vartheta_{i}(t)\right\}$ are the phases, $\left\{\omega_{i}\right\}$ are the natural frequencies, h is a forcing constant and $a_{i,j}$ are constants representing the nonlinear coupling between the ith and jth oscillators. This example is inspired by similar recent studies of [61] and [62], which sought to learn predictive models for the Kuramoto system. Instead, our aim here is to extract structural model information from the system. In particular, we wish to learn the natural frequencies of each oscillator, $\omega_{i}$.

To train our model, we follow [61] and use the kernel

5.3 $$k(u,v)={\left(c+{\left[\begin{array}{c} \sin(u)\\ \cos(u) \end{array}\right]}^{T}\left[\begin{array}{c} \sin(v)\\ \cos(v) \end{array}\right]\right)}^{2},$$

to produce a feature space consisting of constant, linear and quadratic trigonometric terms. This is an example of a kernel with non-polynomial terms, as was discussed in §4d. We seek f that defines the dynamical system 5.2 such that $\dot{x}=f(x)$. The natural frequencies are the constant term in (5.2) so, by §4a, the natural frequencies are approximated by ω≈f(0).

**Figure 7.**
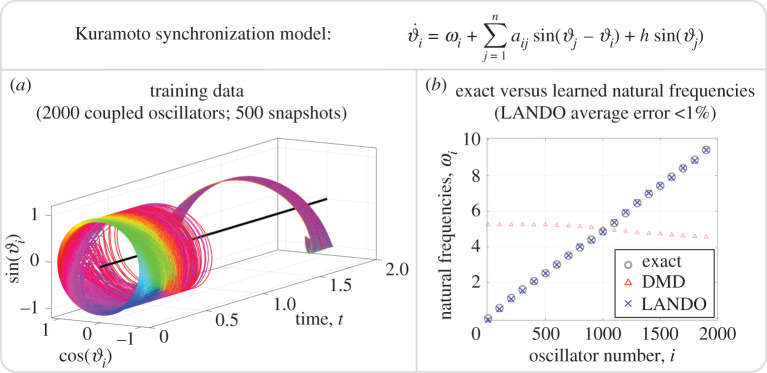
Learning the natural frequencies of coupled oscillators. The training data are generated from a forced Kuramoto model and are illustrated in (*a*). The LANDO framework extracts the natural frequencies of the model. These learned natural frequencies are compared with the true natural frequencies in (*b*) and the frequencies learned by a linear (DMD) model; because there are 2000 oscillators, only a handful of frequencies are plotted. (Online version in colour.)

We consider a system of 2000 coupled oscillators with state vector $x={\left[\vartheta_{1},\;\ldots ,\;\vartheta_{2000}\right]}^{T}$. The data are plotted in figure 7. The feature space, which is implicitly defined by (5.3), has over $2\times 10^{6}$ elements, which is prohibitively expensive to work with explicitly. We consider a strongly coupled system and randomly sample the coupling constants $a_{ij}$ from a normal distribution with mean 15 and variance 5. We take a forcing value of h=2, and randomly sample $\omega_{i}$ from a uniform distribution on the interval [0,10]. The system is integrated to t=2, and we consider only a single simulation.

Figure 7*b* reports the results of the learned natural frequencies. The average error of the estimates made by LANDO is less than 1%. The deviations of the predictions are slightly worse at the upper and lower ends of the spectrum; the cause of this may be that the oscillators synchronize on the average natural frequency, which is 5 here, and the model is more accurate for frequencies close to the average. We also compare the results on a DMD model trained on linear combinations of sin⁡(x), cos⁡(x) and a constant vector. The learned natural frequencies of the linear model are very inaccurate, which illustrates the need of incorporating nonlinearities when attempting to learn the underlying natural frequencies.

### (c) Learning the spectrum of the viscous Burgers’ equation

We now apply our learning framework to study a partial differential equation. The Burgers’ equation is a simplified version of the Navier–Stokes equations and is a prototypical nonlinear hyperbolic PDE. The one-dimensional Burgers’ equation takes the form

5.4 $$u_{t}=\nu u_{xx}-uu_{x},$$

where u(x,t) is the velocity at position x∈[−1,1] and time t≥0, and ν is the kinematic viscosity.

**Figure 8.**
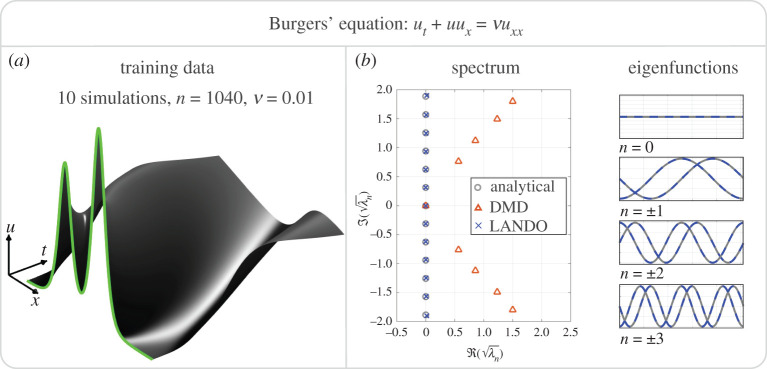
Learning the spectrum of the viscous Burgers’ equation. A typical simulation is illustrated in (*a*) with the initial condition highlighted in green. The algorithm is trained on discrete time snapshots $y_{j}=x_{j+1}$; velocity measurements $\dot{x}$ are not used in the training set. The figures in (*b*) indicate that the algorithm accurately learns the eigenvalues, $\lambda_{n}=-\nu n^{2}\pi^{2}$, and eigenfunctions, $\sin(\lambda_{n}x)$ and $\cos(\lambda_{n}x)$, of the linearized operator at the state u≡0. (Online version in colour.)

We simulate (5.4) with periodic boundary conditions using the spin operator in Chebfun (www.chebfun.org [63]). The solver uses exponential time differencing with fourth-order stiff time-stepping (ETDRK4 [64]). The same method is used to solve the other PDEs in this paper. The kinematic viscosity is ν=0.01 and we use initial conditions

$$u(x,0)=3A_{1}\;{\text{sech}}^{2}(3\sin(\pi (x-2s_{1})))+5A_{2}\;{\text{sech}}^{2}(3\sin(\pi (x-2s_{2}))),$$

where $A_{1,2}$ and $s_{1,2}$ are constants randomly distributed in the interval [0,1]. We perform 10 simulations and integrate to t=1; a typical simulation is shown in figure 8*a*.

We train our model on the state vector defined by the solution u sampled at spatial grid points separated by Δx at time intervals of Δt so that

$$x_{j}={\left[\begin{array}{cccc} u(-1,j\Delta t) & u(-1+\Delta x,j\Delta t) & \cdots & u(1-\Delta x,j\Delta t) \end{array}\right]}^{T}.$$

We use 1024 spatial grid points and take $\Delta t=10^{-3}$. We learn a discrete-time flow map that advances the state vector forward in time by Δt, so $y_{j}=x_{j+1}$. The data are uncorrupted by noise; learning the Burgers’ equation in the presence of noise is explored in the electronic supplementary material, §F.

We use a quadratic kernel to learn a model of this system, and from this model extract a local linearization relative to the equilibrium base state $\bar{x}=0$. The analytical linear operator is simply the Laplacian operator $Au=\nu u_{xx}$. Since the boundary conditions are periodic, the eigenvalues are $\lambda_{n}=-\nu n^{2}\pi^{2}$ for n=0,±1,±2,…. All non-zero eigenvalues have multiplicity two and the eigenfunctions are simply sines and cosines: $\psi_{n}(x)=\sin(\lambda_{n}x),\cos(\lambda_{n}x)$.

The analytical spectrum is compared with that learned by the kernel method in figure 8*b*. The eigenvalues are plotted on a square-root scale so that their spacing is uniform, and the results are compared with the spectrum learned by exact DMD [4]. The present algorithm accurately learns the true spectrum of the underlying linear operator whereas a naive DMD implementation results in substantial errors. The accuracy is best for eigenvalues with larger real part that are associated with slower dynamics, and deteriorates for the eigenvalues associated with quickly dampened modes. Similarly, the kernel method accurately recovers the linear eigenfunctions. The DMD eigenfunctions are very inaccurate and are therefore omitted from the figure.

This example is particularly challenging, as indicated by the poor performance of DMD. The choice of ν=0.01 makes the effect of the linear operator $\nu u_{xx}$ small compared with the nonlinear component $-u_{x}u$. As such, it is particularly difficult for the algorithm to extract the underlying linear operator that is buried beneath nonlinear mechanisms. Additionally, the choice of initial conditions did not provide a particularly rich set of data for the algorithm to work with.

The relatively large size of the state space (approx. $\;10^{3}$) and the high number of samples (approx. $\;10^{4}$) emphasize the necessity of the dimensionality reduction techniques employed in this paper. The kernel trick means that the quadratic feature space need not be constructed explicitly. The dictionary size of this system is approximately 100, which indicates that there are around 100 states that significantly contribute to the underlying dynamics in the high-dimensional feature space. These states are then selected to form the basis of the dynamical model.

This example demonstrates that the algorithm can be used to uncover linear structure highly nonlinear PDEs. We now progress to a more challenging example.

### (d) Learning the spectrum of the KS equation

The KS equation is a PDE that is used to model a range of physical phenomena including turbulence, chemical reactions and flame fronts. The PDE is defined by

$$u_{t}=-u_{xx}-u_{xxxx}-\frac{1}{2}u_{x}^{2},$$

for x∈[−L/2,L/2], periodic boundary conditions and some given initial condition. The KS equation has been described as the ‘simplest chaotic PDE’ [65] and therefore represents a useful test case for our algorithm.

We use our kernel learning algorithm to recover the spectrum of the underlying linear operator relative to the equilibrium state u=0. The linearized operator is $Af=-f_{xx}-f_{xxxx}$. Again, we consider periodic boundary conditions so the eigenvalues are $\lambda_{n}={\left(2n\pi /L\right)}^{2}-{\left(2n\pi /L\right)}^{4}$ with eigenfunctions $\psi_{n}=\sin(\lambda_{n}x)$, $\cos(\lambda_{n}x)$. The initial conditions are now taken to be random periodic functions: in particular, the initial conditions are finite Fourier series with distributed coefficients of equal variance. We define the box length as L=14π, which is sufficiently large to generate chaotic behaviour. The data matrices are constructed in a similar way to that of the Burgers’ equation except we now use velocity measurements in the training data so $y_{j}={\dot{x}}_{j}$. Again, we use 1024 spatial grid points and the samples are separated in time by Δt=0.05. We integrate the PDE to t=60 and use 25 different simulations in the training dataset.

**Figure 9.**
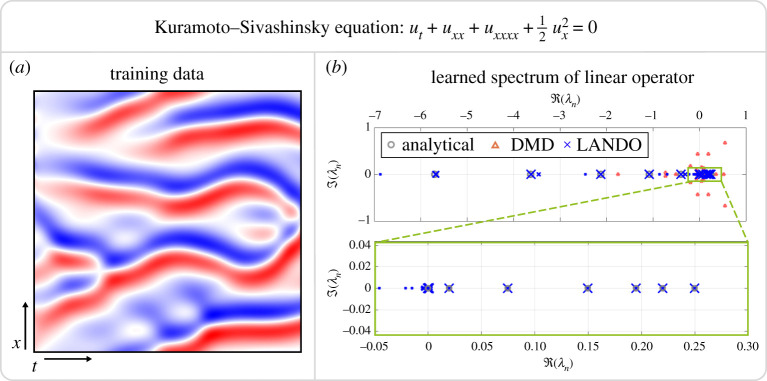
Learning the spectrum of the Kuramoto–Sivashinsky equation with a domain size L=14π, for which the system exhibits chaotic dynamics. A typical simulation is illustrated in (*a*). The algorithm is trained on discrete time snapshots $y_{j}={\dot{x}}_{j}$. (*b*) The algorithm accurately learns the eigenvalues of the linearized operator at the state u≡0. The size of the markers of the LANDO eigenvalues correspond to the average projection of the training data onto the associated eigenvectors. (Online version in colour.)

The results of the learned spectrum are illustrated in figure 9*b*. The algorithm accurately learns the eigenvalues with the correct multiplicity. Similarly to the Burgers’ equation, the recovery of the smallest eigenvalues is most accurate, but the accuracy decreases for eigenvalues with larger negative real part. The close-up figure also indicates that the algorithm recovers the intricate behaviour of the spectrum for small eigenvalues. Although they are not plotted, the eigenfunctions are also recovered to a high degree of accuracy.

This example demonstrates that our implicit learning method can extract accurate information about the underlying linear operator of a chaotic PDE. This performance is encouraging given our eventual goal of studying chaotic, turbulent fluid flows.

## 6. Discussion

We have presented a data-driven kernel method that robustly extracts dynamic modes from high-dimensional, nonlinear data. The method may be viewed as a confluence of DMD, the SINDy and kernel methods. Specifically, we use a kernelized identification of nonlinear dynamics (INDy, i.e. SINDy without the sparsity promoting regularizer) to robustly disambiguate linear and nonlinear dynamics, enabling the extraction of an explicit linear DMD model and forcing snapshot matrix. Access to the disambiguated DMD model and forcing snapshot matrix opens up the possibility of performing data-driven resolvent analysis of strongly nonlinear flows [36]. Our approach is based on the KRLS algorithm [32] and kDMD [7] but introduces several innovations, including stabilized dictionary learning, improved interpretability and extraction of locally linear models and forcing. We have demonstrated our approach on a range of nonlinear dynamical systems and PDEs, and shown in each case that we can effectively disambiguate the roles of linearity and nonlinearity. The nature of kernel methods, along with the online learning variant, render our approach suitable for data that are high-dimensional in both space and time.

### (a) Limitations of the method

There is significant scope for modifications, improvements and generalizations of our framework. In this section, we outline a few key issues; the effects of noise are discussed in detail in the electronic supplementary material, §F.

Our application to the Lorenz system (§5a) demonstrated that the learned linear models depend crucially on the choice of kernel.^3^ A less obvious fact is that, in the underdetermined case, the learned linear model depends on the kernel’s specific hyper-parameters. For example, two quadratic kernels (e.g. $u^{T}v+{\left(u^{T}v\right)}^{2}$ and $2u^{T}v+{\left(u^{T}v\right)}^{2}$) can produce different linear models. This ambiguity stems from the lack of unique solutions to underdetermined systems of equations and emphasizes that kernel hyper-parameters should be selected carefully. These parameters can be chosen via cross-validation, an optimization routine, a hierarchical Bayesian framework [66], or the recently proposed kernel flows [30].

Nonlinear system identification is typically data intensive, and our algorithm is no exception. Our experience indicates that learning an adequate approximation of the linear spectrum of a PDE usually requires a relatively large number of snapshots. For example, we used a space-discretized grid of approximately $\;10^{3}$ points and $10^{4}$ snapshots when learning the viscous Burgers’ equation. One reason for the large number of samples is that the sampling rate must be sufficiently high to resolve the nonlinear dynamics, which may evolve on a faster timescale than the linear component. Since LANDO is generally focused on extracting the linear operator, it needs less data than would be required to identify the full dynamics. However, the difference is not dramatic, and future extensions should reduce the data requirements further by exploiting known physics [67], regularizing the nonlinear component, or employing compressed sensing and random sampling techniques [1].

The right choice of regularizer is essential to the success of any machine learning algorithm. In this work, we used sparse dictionary selection as a regularizer to address the challenges described in §3. However, there are many opportunities to include additional or alternative regularizers within our framework. For example, regularization can be incorporated into the minimization problem (3.5) in a number of ways. The simplest approaches involve modifying the pseudoinverse $k{\left(\tilde{X},X\right)}^{†}$ in the solution (2.16) to incorporate Tikhonov regularization or truncated-SVD regularization.

### (b) Extensions and applications of the method

In addition to the extensions outlined in the previous section, there are many other possible generalizations and applications of our method. This study began with the ultimate aim of performing resolvent analysis [34,35,68] of turbulent flows from a purely data-driven perspective. Over the past decades, advances in numerical methods [69,70] and the growing availability of computational power have enabled analysis of the linearized Navier–Stokes equations for flows of increasing complexity [71]. The authors recently proposed a ‘data-driven resolvent analysis’ [36] based on the DMD, but this approach is currently only applicable for linear flows because strong nonlinearity corrupts the linear DMD model. By separating the roles of linearity and nonlinearity, the present work opens the door to data-driven resolvent analysis of nonlinear and actively controlled PDEs. The ability to perform resolvent analysis in a completely equation-free and adjoint-free manner removes the need to have intrusive access to a numerical solver. We are currently pursuing this approach for low-dimensional PDEs, though we expect that significant modifications to our approach will be needed before we can consider fully turbulent flows.

It is important to note that, in this work, we considered examples with known linearizations, providing a ground truth with which to compare our data-driven linearization. The ground truth is unavailable in practical scenarios, so future work should focus on developing rigorous *a posteriori* methods for validating the LANDO linearization, possibly through cross-validation.

Models that are constrained to respect known physics require fewer training samples and are more robust to noise. In §4d, we explored incorporating partial physical knowledge to design the kernel k, but there is also scope to incorporate known physics into the weight matrix W: efforts are already underway to incorporate such constraints into the learning framework. The LANDO algorithm can be combined with the recently proposed lift and learn framework [72], which uses prior knowledge of a system’s governing equations to construct a coordinate mapping where the dynamics are quadratic. The stabilized dictionary learning step of this work could also reduce the computational cost of the original kDMD algorithm [7].

## Data Availability

Further information is provided in the electronic supplementary material [73]. Data and codes are available at www.github.com/baddoo/LANDO.

## Footnotes

1 This can be thought of as the number of ways of distributing d unlabelled balls into n labelled urns.

2 We use the term ‘sparse’ carefully here. The dictionary is actually a dense matrix but consists of a small number of the total samples. This is the terminology used in the original work of [32].

3 Note that, once the kernel is selected, the linear operator is uniquely specified by condition (4.2).
